# Re-examining the New Product Paradox: How Innovation Ambidexterity Mediates the Market Orientation and New Product Development Performance Relationship

**DOI:** 10.3389/fpsyg.2021.611293

**Published:** 2021-04-27

**Authors:** Anni Zhao, Xinhua Bi, Lei Han

**Affiliations:** Department of Management Science and Engineering, School of Management, Jilin University, Changchun, China

**Keywords:** market orientation, new product development performance, innovation ambidexterity, framework, marketing strategy

## Abstract

More and more well-documented failure of established companies which could not respond to rapid market changes, such as Kodak and Nokia, demonstrate the importance of transferring marketing information into real firm performance. While marketing strategy and management literature has long advocated the direct impact of strong firm market orientation (MO) on new product development (NPD) performance, limited research has discussed the mediating mechanism of this MO-NPD performance relationship. Using the traditional source–position–performance (SPP) framework, this study focuses on the innovation ambidexterity perspective to investigate the mediating mechanism between MO and NPD performance. Then, this study proposed a conceptual framework and propositions to examine the MO - NPD performance relationship further. Theoretical and practical implications of the findings are also discussed.

## Introduction

William Bernbach, the co-founder of Doyle Dane Bernbach (one of the world's largest advertising holding companies), said, “An idea can turn to dust or magic, depending on the talent that rubs against it.” The same is true of the innovation field. Even if these ideas did become real products, they were unable to remain long enough to achieve stable innovation performance. One example of these innovative, yet sadly unsuccessful products would be the Nokia. In contrast with Apple, Nokia missed the smartphone revolution because the company failed to predict what customers wanted. Long after the iPhone's release, Nokia continued to insist that its superior hardware designs and valuable brands would win over users. Even in 2010, Nokia still relied too much on its R&D and research lab, introducing many disappointing phones. Its operating system made matters worse by proving too buggy, clunky, and unintuitive to win consumers over. When companies fall behind, consumers are quick to punish them (Srivastava and Ben-Aaron, 2011). To adapt and survive in today's increasingly dynamic business environment, previous marketing strategy and management literature also suggested that simultaneous exploration and exploitation—experimenting with innovation while improving existing technological capabilities—is prized (e.g., March, 1991; Kortmann, 2015; Zhang et al., 2016). “Innovation is increasingly exploratory the more it departs from knowledge used in prior innovation efforts and, conversely, increasingly exploitative the more deeply anchored it is in existing firm knowledge” (Benner and Tushman, 2003, p. 679). Thus, the case of Nokia and previous literature raised an exciting and imperative research question to answer: Engaging in exploring and exploiting activities, how can firms avoid the new product paradox to build reasonable and sensitive paths to improve innovation performance?

To fill these gaps in the literature, we focus the mechanism between market orientation (MO) on new product development (NPD) performance relationship from the traditional source–position–performance (SPP) framework (Day and Wensley, 1988). We propose that the SPP frameworks help the company gain a competitive advantage by re-examining the black-box from the relationship between MO and NPD performance. After reviewing the previous literature, we involve the strategic perspective to investigate the mechanism of the MO (Responsive and Proactive MO)—NPD performance relationship.

Research on “organizational ambidexterity” has well recognized the benefits to firm performance when keeping a closer balance between exploration and exploitation within organizations (e.g., Gibson and Birkinshaw, 2004; He and Wong, 2004; Gupta et al., 2006; Cao et al., 2009; Uotila et al., 2009; Heavey and Simsek, 2017; Koryak et al., 2018). Explorative capability refers to a firm's ability to identify and assimilate new scientific and technological knowledge. In contrast, exploitative capability refers to a firm's ability to transform and apply their existing knowledge and resources (March, 1991; He and Wong, 2004; Atuahene-Gima, 2005; Yalcinkaya et al., 2007; Lisboa et al., 2011). By involving explorative innovation, firms may take advantage of their existing resources to identify new technological opportunities and develop new products (Zhang et al., 2016). By pursuing exploitative innovation, firms may explore knowledge variants and develop new capabilities to “replace inefficient capabilities with more efficient ones” (Sirmon et al., 2011, p. 1402) to seek out future technology and market opportunities (Zhang et al., 2016). Following the traditional source–position–performance (SPP) framework, firms' strategic orientations (e.g., market orientation) play a crucial *source* role in driving innovation and “represent the ability of a business to do more or do better (or both) than its competitors” (Day and Wensley, 1988, p. 2), especially by the ambidextrous pursuit of both/or exploitative and explorative innovation (Zhang et al., 2016; Ghantous and Alnawas, 2020). Such source advantage may not automatically convert into sustainable performance. By adopting exploitative and/or explorative innovation, innovation ambidexterity works as the strategic perspective, creating a unique *position* advantage to transfer their knowledge and resources into performance. And Such a strategic perspective means the management and organizational capability to both compete in a mature market and to expand new products and services in an emerging market (Tushman and O'Reilly, 1996). This study contributes to the marketing/management literature and practices in several ways. First, we extend Day and Wensley's (1988) SPP framework by further checking and exploring the mechanism between market orientation and NPD performance. Specifically, by adopting this classical SPP framework into the innovation context, we initially examined the important mediated role played by innovation ambidexterity between MO and NPD performance. Moreover, we extend the prior knowledge on the traditional market orientation and generate a conceptual framework to explore the relationship between responsive and proactive MO and NPD performance, mediated by innovation ambidexterity. Besides, we also proposed that the differential effects of responsive and proactive MO on NPD performance, which paves the way for future empirical studies on this research field. Finally, a good strategy can lead to detailed and tactical execution. Therefore, this research may guide managers about where to allocate their precious resources and efforts to improve their competitive advantage and NPD performance.

## Literature Review of Market Orientation (MO) and New Product Development (NPD) Performance

### Market Orientation (MO)

Market orientation is a set of capabilities that establishes the customer's current and future needs first and then is focused on generating, disseminating, and using the information about customers and competitors to create superior value for the customers (Kohli and Jaworski, 1990; Narver and Slater, 1990). Deshpandé et al. (1993) only focused on the “customer orientation as the set of beliefs that puts the customer's interest first, while not excluding those of all other stakeholders such as owners, managers, and employees, to develop a long-term profitable enterprise” (p. 27). They found that self-reference and customer assessments of customer orientations are uncorrelated. Hence, the customer perspective serves as a reality check (Deshpandé et al., 1993). To avoid firms that may overemphasize their existing customers' needs and limit their innovative activities, Narver et al. (2004) suggested a more comprehensive and holistic approach to customer needs and solutions perspective, redefining another two types of MO: *a responsive market orientation* and *a proactive market orientation*. In this study, following Narver and his colleagues' new classification, we defined the *responsive market orientation* as the discovery of the customer's needs and solutions of which the customer is aware, while the *proactive market orientation* is the exploration of needs and solutions of which the customer is unaware.

As we can see from the above MO definition, it is essential to note that MO is conceptually and operationally different from organizational proficiency in performing marketing-related activities or market orientation in specific events, such as new product development field. In the following section, we will summarize the previous research findings related to the relationship between MO and NPD performance.

### Literature Review for the MO–NPD Performance Relationship

The previous literature has generalled that the new product success can be divided into different important antecedents, such as the competitive environment, the internal environment, or the new product development process (Song and Parry, 1997). In the following section, we focus on most of these factors to involve market orientation as the critical antecedent variables for NPD performance and summarize the previous research findings related to MO and NPD performance.

Atuahene-Gima (1995) presented the research question by observing how MO influences new product performance, and empirically tested that MO has a significant positive effect on new product performance and new product development activities. Song and Parry (1997) also found that firms in touch with the market may have a better understanding of customer wants and needs, competitor activities, and market trends, which can lead to better new product success. Wren et al. (2000) evaluated the impact that MO has on new product success in a cross-national context and found that MO is essential to new product success. By extending the measurement of both responsive market orientation and proactive market orientation, Narver et al. (2004) found that a responsive MO is not sufficient and, thus, that a proactive MO plays a vital positive role in a business's new-product success. The relevant literature on responsive market orientation and proactive market orientation in the fields of management and marketing field are summarized in Table 1.

**Table 1:** A chronological review of the literature on responsive market orientation and proactive market orientation.

**References**	**Context**	**Independent variables**	**Mediated variables**	**Moderator variables**	**Dependent variables**	**Key findings**
Narver et al. (2004)	41 technologically business units from 25 companies	Responsive MO; Proactive MO; Innovation Orientation			New product success	An RMO is not sufficient and, thus, that a PMO plays a very important positive role in a business's new-product success.
Atuahene-Gima et al. (2005)	175 U.S. firms	Responsive MO; Proactive MO		Strategic consensus; Learning orientation; Marketing's power in the firm	New product performance	RMO has a U-shaped relationship with NPD, PMO has an inverted U-shaped relationship with NPD; Joint effect has negatively related to NPD; strategic consensus (+) moderated responsive MO-NPD; learning orientation and marketing's power (+) moderated proactive MO-NPD.
Tsai et al. (2008)	384 public high-tech firm in Taiwan	Responsive MO; Proactive MO		Competitive intensity; technological turbulence	New product performance	Both PMO and RMO positively related to NPD; PMO has an inverted U-shaped relationship with NPD; TT (-) moderated curvilinear RMO-NPD; CI (+) moderated curvilinear PMO-NPD.
Voola and O'Cass (2010)	189 Australia firm	Differentiation; cost leadership	Responsive MO; Proactive MO		Firm performance	Both competitive strategies influence RMO and PMO and firm performance. However, the results show that differentiation strategy has a stronger influence on RMO and PMO than cost-leadership strategy, and that PMO has a stronger influence on performance than RMO.
Bodlaj (2010)	325 Slovenian companies	Responsive MO; Proactive MO	Degree of novelty		Firm performance	Only a PMO is positively related to the degree of novelty.
Herhausen (2016)	167 two waves survey data	Responsive MO; Proactive MO			Market performance	Only PMO positively related to NPD; the balance between proactive and responsive market orientation has an incremental positive effect on performance beyond their combined effect; that performance will decline less sharply when proactive is higher than responsive market orientation; and that as the level of balance increases, performance will first decrease and then increase.
Jaeger et al. (2016)	Panel data of 56 US companies observed over 9 years	Responsive MO; Proactive MO			Firm performance	PMO has a U-shaped relationship with preperformance, RMO has an inverted U-shaped relationship with preperformance; the results confirm the proposed inverted-U-shaped carry-over effects of RMO, PMO exerts no carry-over effects.

So far, there are two major meta-analyses related to MO and NPD performance in marketing literature. Kirca et al. (2005) provided a summary of the bivariate findings regarding the antecedents and the consequences of MO. For the relationship between market orientation and innovation consequences, they found that market orientation has positive associations with both an organization's innovativeness (*r* = 0.45, *p* < 0.05) and new product performance (*r* = 0.36, *p* < 0.05). Grinstein (2008) found that overall market orientation is positively related to innovation consequences (*r* = 0.396, 95% of CI: 0.352–0.440). In addition, they also found that the three dimensions of MO has a positive effect to innovation consequences separately, customer orientation-innovation consequences (*r* = 0.411, 95% of CI: 0.339–0.483), competitor orientation-innovation consequences (*r* = 0.373, 95% of CI: 0.268–0.478), and inter-functional coordination-innovation consequences (*r* = 0.392, 95% of CI: 0.295–0.488).

Although most of the previous literature supports the positive relationship between MO and NPD performance (Wei and Morgan, 2004), there are some different opinions regarding the positive MO-NPD performance relationship. For example, some scholars argue that strict adherence to the tenets of the marketing concept philosophy leads to more miserable innovation activities and performance in the long run (Hayes and Abernathy, 1980). Also, empirical research provides inconclusive evidence on the influence of MO on new product development activities. For example, Baker and Sinkula (2009) found that MO does not influence firm innovation performance (profitability) directly, but entrepreneurial orientation (EO) does, so they concluded that market orientation and entrepreneurial orientation have a complementary effect on profitability in small businesses. What's more, in our in-depth interviews with some senior managers, we had the same dilemma that it is very difficult to earn superior innovation performance from their kinds of “innovative product.” The environment is changing so quickly that the new product that costs lots of resources to develop is easily out of date, even before launching the product in the market. Therefore, in the following section, we try to re-investigate the mechanism between MO and NPD performance to figure out the black-box between the MO-NPD performance relationship.

### Re-examining the MO-NPD Performance Relationship

The SPP model helps the firm develop a competitive advantage by using its resources and competencies. The basic ideas of the SPP model come out the sequential determinism of competitive advantage: the source → position → performance, which states that the source of firm or industry determines the position of firms (including their room for product differentiation or lowest delivered cost position), ultimately, in turn, determines their performance (including customer satisfaction, loyalty, market share, and profitability) (Day and Wensley, 1988). This framework provides a useful perspective for examining the relationship between MO and NPD performance. Adapting the above SPP model, Im and Workman (2004) found that new products (NP) and related marketing programs (MP, such as packaging, warranties, pricing, promotion, distribution) creativity mediates the relationship between market orientation and new product success. Langerak et al. (2004) tried to investigate the relationship among MO → new product advantage/the proficiency in new product launch activities → new product performance → organizational performance.

In addition to these studies listed above, more studies are needed to explore the mechanism between MO and NPD performance. To address the research question of re-examining the black box between MO and NPD performance, we also employ Day and Wensley's (1988) source—position—performance (SPP) framework for our conceptual model. Following the previous literature adopted the SPP framework in the marketing, management, and innovation literature (Im and Workman, 2004; Zhao et al., 2015), we believe such SPP framework is appropriate for examining the relationship between MO, innovation ambidexterity, and firm's NPD performance. Specifically, we propose that MO (responsive and proactive MO) is a necessary, but not sufficient prerequisite for high firm performance. For achieving superior performance, the innovative firms must successfully “represent the ability of a business to do more or do better (or both) than its competitors” (Day and Wensley, 1988, p. 2), and develop and foster the right mix of innovation capabilities (i.e., exploitative and explorative innovation) to utilize their existing or new innovative capabilities to transfer their knowledge and resources into performance. In the following section, based on the SPP framework, we introduce the related constructs and develop the propositions in our conceptual model.

### The Mediation Effect of Innovation Ambidexterity on the MO-NPD Performance Relationship

March (1991) defined that exploration requires “search, variation, experimentation, play, flexibility, discovery, and innovation,” and exploitation requires “refinements, choice, production, efficiency, selection, implementation, and execution” (p. 71). Prior research builds on these perspectives and focuses on innovation ambidexterity, which is defined as a firm's ability to concurrently develop explorative and exploitative capabilities for innovation (Tushman and O'Reilly, 1996; He and Wong, 2004; Fernhaber and Patel, 2012; Lin et al., 2013). By involving exploitative innovation, firms may take advantage of their existing resources to identify new technological opportunities and develop new products (Zhang et al., 2016). By pursuing explorative innovation, firms may explore knowledge variants and develop new capabilities to “replace inefficient capabilities with more efficient ones” (Sirmon et al., 2011, p. 1402) to seek out future technology and market opportunities (Zhang et al., 2016). According to the previous literature, “MO is related to a firm's overall value and business philosophy about the importance of serving customers' needs” (Fang and Zou, 2009, p. 744), while, innovation ambidexterity (i.e., exploitative and explorative innovation) emphasize marketing process adjustment capabilities or activities in response to market changes and help a company respond to environmental changes through marketing and technological adaptation. Such adaptations are facilitated by processes such as resource integration, organizational learning, and knowledge management, enabling firms to maintain a sustainable advantage and achieve superior performance over their competitors (Xu et al., 2018). Since there are two distinctive types of MO—responsive and proactive market orientation (Narver et al., 2004), in the following section, we turn our attention to investigate the effect of how responsive and proactive MO can facilitate innovation ambidexterity, ultimately increase firm new product development performance.

*Exploitative innovation*. In this research, we define exploitative innovation as the firms' capability that entails a shift away from its *existing* technological knowledge base, such that it enables the firm to enter new product-market domains. As we discussed above, exploitative innovation is an innovation that leverages current skills and capabilities, which enables the firm to improve existing product-market positions (Lavie et al., 2010). Exploitative innovation builds on existing marketing and technological knowledge and reinforces existing skills and processes (Lewin et al., 1999; Benner and Tushman, 2003). Moreover, the prior study has suggested that firms must pursue a customer-oriented philosophy and strengthen their marketing and sales skills to succeed in pursuing exploitative innovation (Atuahene-Gima, 2005). To boost such exploitative innovation, we propose that both responsive and proactive market orientation are essential.

As for the responsive market orientation, similar to the traditional measures of market orientation to discover the needs and solutions of a customer of which the customer is aware and can express, it has been approved as an essential factor in successful new product development (Atuahene-Gima, 1995; Atuahene-Gima and Ko, 2001; Narver et al., 2004). Following this logic, these firms are identified and committed to the organization's responsive customer-oriented value, such as understanding and serving the needs of current customers; they may excel in searching for their existing resources and using market information to serve the customer better (Day, 1994). Besides that, by leveraging their customer knowledge, they can become aware of market opportunities and improve their existing processes and resources to better satisfy existing customer needs (Yalcinkaya et al., 2007). In sum, by hearing customer's voices and adapting current offerings, responsive market orientation can help the company to foster exploit innovation opportunities, which allows them to take advantage of their existing learning and experience to address the customer's expressed needs; ultimately improve the firm performance (Ghantous and Alnawas, 2020).

Proactive market orientation aims to explore the needs and solutions of which the customer is unaware (Narver et al., 2004). A firm with a high proactive market orientation could entail the research for new and diverse market information fit for the firms' existing scope of knowledge and experience. By leveraging their existing resources and diverse marketing information, proactive firms may find and satisfy consumers' latent needs and guide the firm to emphasize the experimentation into their organizational activities. Focusing on future customer needs can also provide information to those firms about new markets and technology advancements to improve companies' capabilities to integrate existing resources into product innovation. Thus, we make the proposition that both responsive market orientation and proactive market orientation directly benefit an exploitative innovation, ultimately help to improve the NPD performance:

*P1a: Exploitative innovation mediates the relationship between responsive MO and NPD performance*.
*P1b: Exploitative innovation mediates the relationship between proactive MO and NPD performance*.

*Explorative innovation*. In this research, explorative innovation refers to firms' ability to identify and assimilate *new* scientific and technological knowledge. According to the definition, explorative innovation attempts to adopt new processes, products, and services that are unique from those used in the past (Levinthal and March, 1993; Yalcinkaya et al., 2007). Moreover, such explorative innovation requires new technological and market knowledge that departs from the firm's existing knowledge-base to better match the environment (Eisenhardt and Martin, 2000; Balboni et al., 2019; Wang et al., 2019). We also posit that both responsive and proactive market orientation are critical to boosting explorative innovation.

“Rome was not built in a day.” Such responsive market-oriented firms not only respond to existing customer needs but also may uncover latent needs and anticipates future needs (Narver and Slater, 1990; Day, 1994). Thus, such firms may be eager to build on new capabilities (exploration), as existing ones (exploitation) become inadequate. When developing new technologies, those firms also can identify with their current customers who are appropriate for evaluating the products developed with those brand new technologies to ensure their success (Yalcinkaya et al., 2007). Besides, a firm with high responsive market orientation may deeply understand their current knowledge and experience resources, which may better able to integrate their existing knowledge to solve customers' problems and reduce the risks of exploration (Atuahene-Gima et al., 2005).

Sometimes, innovation is not just limited to slightly changing the existing product, but also takes the form of big ideas, means, or objects perceived by a person as something radically new. Through this track, innovation refers to the products which have not existed in the market in this period. In this situation, proactive market-oriented firms may be more associated with radical innovation than a responsive market orientation, which focuses on the company's current knowledge and experience to explore new technologies. Previous literature also suggested that firms with proactive market orientation enable the firm to nurture creative ideas and improves the firm's problem-solving capacity, thus advancing the effectiveness of exploration (Atuahene-Gima et al., 2005). Thus, we make the proposition that both responsive market orientation and proactive market orientation may benefit from an explorative innovation:

*P2a: Explorative innovation mediates the relationship between responsive MO and NPD performance*.
*P2b: Explorative innovation mediates the relationship between proactive MO and NPD performance*.

### The Relative Effect of Market Orientation (Responsive and Proactive MO) on the Innovation Ambidexterity

The knowledge about the business's customers and competitors could lead to more effectively market targeting, product development, and positioning (Hunt and Morgan, 1995; Atuahene-Gima and Ko, 2001). Proactive market orientation involves organizational processes for learning about the latent needs of current or potential customers, which may help the company adapt quickly to market shifts (Herhausen, 2016). In addition, proactive market orientation seems to foster the company to adopt some risky and fruitful strategies to lead and educate customers' latent needs. Apple can be the perfect example to show the power of proactive market orientation on explorative innovations. No customer gave Steve Jobs and Apple the design for the iPhone or the iPad. Instead of that, they came about from intense listening combined with a creative leap within their engineers to tackle and train customers' perceived needs (Guo et al., 2018). Therefore, proactive market orientation is more important to develop new products (explorative innovations) than responsive market orientation. In support of this argument, Narver et al. (2004) find that although both proactive and responsive market orientations are positively related to innovation, proactive market orientation may have a stronger effect than responsive market orientations. Consist of these empirical findings, another empirical paper also found that a (responsive) market orientation is not necessarily limited to incremental innovation (Baker and Sinkula, 2009).

As for the responsive market orientation, there are lots of business cases to prove in their efforts to satisfy customers' current needs. For example, Walmart, as one of the most efficient logistics companies, by continuously improving its physical handling and distribution processes (exploitative innovation), can seek to create superior value for customers primarily interested in low prices to satisfy their current needs. Previous literature also suggested that a responsive market orientation is viewed as contributing mainly to customer-led innovations but very little to major innovations (Yalcinkaya et al., 2007). Therefore, we propose that the responsive MO could more consistent with an exploitative learning strategy and have a more substantial effect on exploitative innovation than proactive MO. Thus, we make the following proposition:

*P3a: The effect of proactive MO on explorative innovations is stronger than responsive MO*
*P3b: The effect of responsive MO on exploitative innovation is stronger than proactive MO*

## Discussion

Drawing upon Day and Wensley (1988) classical SPP framework, we initially explore and investigate the mediating mechanism between market orientation and new product development (NPD) performance. More specifically, we propose that market orientation is a necessary, but not sufficient prerequisite for high firm performance. To maintain competitive advantages and achieve superior performance, previous research has empirically examined performance outcomes in general (e.g., innovation, profitability) by matching between a particular culture and other marketing mix factors to utilize their existing or new innovative capabilities to transfer their knowledge and resources into performance (Zhang et al., 2016). Therefore, we gain insight into extending the traditional market orientation into the proactive and responsive market orientation perspective to explore whether and how they have the mediating effects of fostering innovation ambidexterity, ultimately improving their NPD performance.

The findings of our conceptual model proposed that exploitative innovation mediates the relationship between MO (responsive MO and proactive MO) and NPD performance, and the explorative innovation also mediates the relationship between MO (responsive MO and proactive MO) and NPD performance. In addition, we also proposed that exploitative innovation and explorative innovation may have differential effects on the relationship between responsive and proactive MO and NPD performance. More specifically, the effect of proactive MO on explorative innovations is stronger than responsive MO, while the effect of responsive MO on exploitative innovation is stronger than proactive MO. Next, we discuss the findings' implications to theory and managerial practice and the limitations and opportunities for future potential research.

## Theoretical Implications

This research contributes to the marketing/management literature in the following approaches. First, we extend Day and Wensley's (1988) SPP framework by further checking and exploring the mediating mechanism between MO and NPD performance. Besides that, by adopting this classical SPP framework into the innovation context, we initially examined the important mediated role played by innovation ambidexterity between MO and NPD performance.

Furthermore, we extend traditional market orientation literature into two different angles- response MO and proactive MO to generate a conceptual framework to explore the relationship between responsive/proactive MO and NPD performance, mediated by innovation ambidexterity. Moreover, we also proposed that the differential effects of responsive/proactive MO to gain insight into how to foster the innovation ambidexterity, ultimately improve NPD performance. Such a conceptual model and propositions may pave the way for future empirical studies on this research field.

## Managerial Implications

Our findings provide important implications for marketing managers. First, it can give marketing managers a new strategic direction to achieve competitive advantages and superior performance, especially for achieving new product development performance. The marketing managers should focus on both the “*responsive and proactive market orientation*” approach, enabling the business to compete by anticipating market requirements ahead of competitors and creating long-lasting relationships with customers, channel members, and suppliers (Gulati, 1999).

Also, there is an essential mechanism for giving marketing managers knowledge to transform responsive and proactive market orientation to new product development performance. Managers can spot opportunities and threats before rivals to guide managers on where to allocate their precious resources and efforts to balance with exploratory and exploitative innovations (Dai et al., 2017).

## Limitations and Future Research

Several caveats bound the implications of our results, which offer interesting future research opportunities. First, this research only focuses on the mediating mechanism between MO and NPD performance. Some important antecedent variables for MO may be inevitably excluded in this research. Another limitation of this study is that this is a conceptual piece of a framework to explore the mediating mechanism between MO and NPD performance, more theoretical evaluation and different perspectives (RBV or KBV) may be considered to examine the dynamic nature of the relationships we have discussed.

Second, this study only considered the strategic (innovation ambidexterity) perspective to investigate the mediating mechanism between MO and NPD performance, and other mechanisms (e.g., marketing capability, internal social capital integration, etc.) could be used in future research. At the same time, a key area for theory development would be to study what may influence the co-existence (joint effect) or strength of the direct effects we have examined.

Finally, this study only took the bright side of MO. In the future, an attempt to find some boundary conditions, and the empirical test will be interested in the marketing or management discipline. For example, organizational factors (e.g., organizational connectivity), industry and environmental factors (e.g., perceived environmental uncertainty), or cultural settings (e.g., individualism and collectivism) can be brought to bear as potential moderators.

## Author Contributions

AZ, XB, and LH: conceptualization. AZ: writing—original draft preparation and writing—review and editing.

## Conflict of Interest

The authors declare that the research was conducted in the absence of any commercial or financial relationships that could be construed as a potential conflict of interest.
